# Spatial proximity between T and PD-L1 expressing cells as a prognostic biomarker for oropharyngeal squamous cell carcinoma

**DOI:** 10.1038/s41416-019-0634-z

**Published:** 2019-12-06

**Authors:** Anna Maria Tsakiroglou, Martin Fergie, Ken Oguejiofor, Kim Linton, David Thomson, Peter L. Stern, Susan Astley, Richard Byers, Catharine M. L. West

**Affiliations:** 1 Division of Cancer Sciences, University of Manchester, Manchester, M13 9PG UK; 2 Division of Informatics, Imaging and Data Science, University of Manchester, Manchester, M13 9PG UK; 3 Dorset Cancer Centre, Poole Hospital NHS Foundation Trust, Poole, BH15 2JB UK; 4 The Christie NHS Foundation Trust, Manchester, M20 4BX UK; 5 Manchester Cancer Research Centre, University of Manchester, Manchester, M20 4GJ UK; 6 Division of Informatics, Imaging and Data Science, Manchester Breast Centre, Manchester Cancer Research Centre, University of Manchester, M13 9PG Manchester, UK; 7 Division of Cancer Sciences, University of Manchester, Manchester Royal Infirmary, Manchester, M13 9WL UK; 8 Division of Cancer Sciences, University of Manchester, The Christie NHS Foundation Trust, Manchester, M20 4BX UK

**Keywords:** Image processing, Prognostic markers, Cancer imaging, Head and neck cancer, Cancer microenvironment

## Abstract

**Background:**

Fulfilling the promise of cancer immunotherapy requires novel predictive biomarkers to characterise the host immune microenvironment. Deciphering the complexity of immune cell interactions requires an automated multiplex approach to histological analysis of tumour sections. We tested a new automatic approach to select tissue and quantify the frequencies of cell-cell spatial interactions occurring in the PD1/PD-L1 pathway, hypothesised to reflect immune escape in oropharyngeal squamous cell carcinoma (OPSCC).

**Methods:**

Single sections of diagnostic biopsies from 72 OPSCC patients were stained using multiplex immunofluorescence (CD8, PD1, PD-L1, CD68). Following multispectral scanning and automated regions-of-interest selection, the Hypothesised Interaction Distribution (HID) method quantified spatial proximity between cells. Method applicability was tested by investigating the prognostic significance of co-localised cells (within 30 μm) in patients stratified by HPV status.

**Results:**

High frequencies of proximal CD8^+^ and PD-L1^+^ (HR 2.95, *p* = 0.025) and PD1^+^ and PD-L1^+^ (HR 2.64, *p* = 0.042) cells were prognostic for poor overall survival in patients with HPV negative OPSCC (*n* = 31).

**Conclusion:**

The HID method can quantify spatial interactions considered to reflect immune escape and generate prognostic information in OPSCC. The new automated approach is ready to test in additional cohorts and its applicability should be explored in research and clinical studies.

## Introduction

It is recognised that a plethora of immune regulatory factors in the tumour microenvironment (TME) contribute to the progression of cancers and limit their response to treatment.^1–3^ An important class of inhibitory factors, designated immune checkpoints, have been associated with sustained tumour responses in a variety of cancers.^4,5^ The programmed cell death 1 (PD-1) receptor has emerged as a dominant negative regulator of anti-tumour effector function. Interaction with its ligand PD-L1 leads to PD-1 mediated T cell exhaustion and inhibition of antitumour cytotoxic T cells. The latter results from specific T cells releasing interferon gamma (IFN-γ^+^) after recognising their tumour associated antigens. IFN-γ^+^ release leads to upregulation of PD-L1 on the local tumour and other cells, which in turn can compromise T cell function through adaptive immune resistance. This state of local immune privilege can be reversed by blocking antibodies to PD-1 or PD-L1 and such single agent therapies are now licensed for the treatment of patients with multiple types of cancers.^4–10^ Response rates can be as high as 90% for some tumour types but as low as 15% with others, but selection of patients likely to respond favourably to such single agent therapy proves a challenge, as it requires an in depth understanding of immune interactions in the TME.^4,5^

Head and neck squamous cell carcinomas (HNSCC) develop as a consequence of either a persistent high risk HPV infection or through carcinogen exposure (e.g. smoking, alcohol).^11^ In the subgroup of oropharyngeal squamous cell carcinomas (OPSCC), the HPV positive patients have a significantly better clinical outcome and this is linked to differences in tumour infiltrating lymphocyte (TIL) densities.^12^ PD-L1 positivity within a tumour has been explored as a potential treatment biomarker but the results have not been consistent in predicting subsequent clinical responses.^13–17^ The spectrum of “conclusions” may not be surprising considering the variability of tumour aetiology, the antibodies and detection methodologies used, the arbitrary cut-off levels defined and cellular diversity of cells expressing PD-L1. Moving beyond simple enumeration of cell densities, and observing the spatial organisation of the TME may provide further insight for the development of more informative biomarkers.^18,19^ In the TME of HNSCC the existence of varying patterns of PD-L1 expression has been highlighted.^20^ These qualitative results are useful pointers to further analysis but are not easily generalised, as the criteria for defining patterns are subjective. Quantitative, non-subjective, assessment of spatial organisation becomes possible using automated image analysis approaches^15,21^ to minimise operator dependence and analysis time, and facilitate successful clinical application.

Here we report an automated analysis pipeline to quantify the potential of T cells to interact with PD-L1 expressing cells in the TME, which will reflect a key driving force for immune regulation. Our algorithm discards artefacts and scanning errors, performs cell segmentation and accounts for the proximity between cell subsets. Using the Hypothesised Interaction Distribution (HID) method^22^ we assess whether a high frequency of spatial interactions between CD8^+^ or PD-1^+^ and PD-L1^+^ cells correlates with a poor prognosis in OPSCC, as previously observed in HPV^-^ OSCC.^21^

## Materials and methods

### Cohort characteristics

The dataset for this study derived from a retrospective collection of 218 OPSCC patients treated with radiotherapy alone or with concurrent chemotherapy at The Christie NHS Foundation Trust in Manchester, UK between January 2002 and December 2011 (REC reference: 03/TG/076). HPV status of these patients was assessed (p16 expression, in-situ hybridisation and human papillomavirus DNA PCR) as described elsewhere.^12^ Within this cohort, 124 patients with concordant HPV status for all three assays had sufficient formalin fixed, paraffin embedded tissue available for multiplex immunofluorescence staining with antibodies against PD-L1, CD8, CD68 and PD-1.^15^ Analyses were performed on randomly selected regions of interest (ROIs) from sections taken from pre-treatment diagnostic biopsies of OPSCC. The associated clinical data for grade, stage and comorbidities (alcohol and smoking) is described elsewhere.^15^ Updated overall survival (OS) information was obtained for 72 patients.

### Multiplex staining and multispectral scanning

Multiplex immunofluorescent staining was performed using the Ventana auto-staining platform (Ventana Medical Systems, Oro Valley, Arizona, United States) and the Opal detection system (PerkinElmer, Waltham, Massachusetts, United States) with tyramide signal amplification (TSA), as described elsewhere^16^ and summarised in Supplementary Table 1. Using TSA^23^ and the Opal kit technology permits multiple repeated cycles of staining and stripping of anti-mouse or anti-rabbit antibodies, while the TSA conjugated fluorophores bind strongly to the epitopes and remain on the tissue. The auto-staining platform performed an initial deparaffinisation and epitope retrieval at pH 8.5. Subsequent staining cycles involved incubation with the primary antibody, the secondary antibody, and then the opal detection label. Each staining cycle was separated by a short denaturation at pH 6. After staining, slides were washed with EZ preparation (1:10) for three cycles of 5 min each and cover-slipped using the Prolong aqueous mounting agent (Thermo Fisher, Waltham, Massachusetts, United States) with DAPI for counter-staining. Imaging was performed using a Vectra microscope (PerkinElmer) and a 20x objective (0.495 µm per pixel). The Vectra microscope first scanned whole slides at low resolution to obtain the tissue grid using only the DAPI filter. Subsequently, 10–20 ROIs (1392 × 1040 pixels) were selected randomly for each slide from tissue areas for multispectral scanning at full resolution using all available filters (DAPI, FITC, Cy3, Texas Red and Cy 5).

### Spectral un-mixing

Linear spectral un-mixing^24^ was performed using the inForm software (PerkinElmer). For un-mixing a spectral library was built comprising individual fluorophore spectra. Each spectrum was acquired from slides that were single stained for the different antibodies, using the same experimental parameters as in the multiplex experiment. A slide stained only with DAPI was also used to extract the DAPI spectrum. Finally, a slide that underwent all steps in the multiplex experiment without application of antibodies or fluorophores was used to extract the spectrum of tissue auto-fluorescence (AF). After spectral un-mixing, the images had 6 channels (1392×1040×6 pixels), each containing the intensities of a different fluorophore (see Supplementary Fig. 1).

### Deep learning for automated identification and exclusion of problematic areas

After spectral un-mixing, a quality assessment of images was needed to verify that only relevant areas of tissue were included in subsequent analyses. To discard artefacts and select areas of tissue suitable for analysis, supervised tissue segmentation using support vector machines (SVM) or convolutional neural networks (CNN) has previously been employed successfully.^25,26^ We show that the CNN approach can also be used with immunofluorescence, where apart from blurring and artefacts, high auto-fluorescence in blood vessels and red blood cells cause problems. Immunofluorescence image artefacts include: bubbles created during cover-slipping; tissue folding; blurriness due to scanning errors; the presence of blood vessels with brightly auto-fluorescing red blood cells; and the presence of fatty tissue. Digital pathology datasets tend to be large, making manual checking of images to identify and exclude problematic areas slow and labour intensive.

To automate this essential pre-processing step, a deep CNN classifier was trained on a set of 3280 manually annotated image patches of size 128 × 128 × 6 to discriminate at pixel level between problematic areas, useful tissue, or background. The image undergoes a series of transformations as it passes through the layers of the network and a predicted output label is generated for each pixel. This output is compared to the ground truth and the parameters of the network are updated during training to decrease the error. A variant of the U-Net network architecture,^27^ popular in biomedical applications, was used as detailed in Supplementary Fig. 2. Additional implementation details are given in Supplementary Material 2.

A test set of 640 images was used to assess performance. Pixel-wise accuracy was 88.3% when compared with manual annotations (Supplementary Fig. 3 and Supplementary Table 2). For comparison, a tissue segmenting module trained using inForm 2.4 software to perform the same task on the same training set achieved an accuracy of only 81.2% on the test set (implementation details in Supplementary Material 2). Therefore, the CNN was applied to remove artefacts and background. If there was <30% useful tissue identified by the CNN the ROI was excluded from subsequent analysis. After pre-processing, the dataset was reduced to 1620 images (1392 × 1040 × 6 pixels), and only cells with centroids located within the useful tissue areas were considered for subsequent analyses.

### Cell segmentation and scoring

Cell segmentation was carried out using the open source digital pathology software QuPath v0.1.3.^28^ Nuclear detection was performed on the DAPI channel using an unsupervised watershed algorithm with parameters tuned on a validation set of 10 ROI. QuPath’s nuclear detection algorithm was quantitatively tested in a separate test set of 5 manually annotated ROI and its performance was found equivalent to that of the commercial software inForm 2.4 in terms of cell-wise average precision, as in ref. ^29^ (see Supplementary Material 3, Supplementary Tables 3 and 4). While both software packages produce similar results as seen in Supplementary Fig. 4, QuPath was selected for this study as it is open-source software, with well-maintained documentation, version management and an active supportive community. Furthermore, it offers built-in capability of custom scripting, which facilitates quantitative validation of its algorithms’ performance. After nuclear detection, the cytoplasm around each nucleus was simulated by cell expansion of 2 μm and measurements generated for marker intensity in different compartments (mean, minimum, maximum and standard deviation of intensity in cytoplasm or nucleus). Details of this procedure are shown in Supplementary Fig. 5.

Positivity was determined by the intensity of each marker in the primary cell compartment where it is usually expressed. In our study, markers were cytoplasmic or membranous. Before cell scoring, the intensity of each marker was re-scaled onto a grey-scale colour map, with the brightest and darkest values corresponding to the 99% and 1% percentiles of the marker’s pixel intensities in the entire dataset. Having a consistent colour-map per marker ensured that the same intensity value was represented with equal brightness in all images.

Guided by a pathologist (R.B), a single threshold for each marker was selected as a cut-off to determine positivity across the entire dataset. The threshold was identified by its ability to separate positive from negative cells in a set of 20 ROIs from 20 different patients (Supplementary Fig. 6). This cell scoring method was chosen for its simplicity but provided a non-optimal separation in some samples, possibly due to slight variations in fixation, staining, scanning or cell segmentation performance. For subsequent analysis these small variations were ignored, however their presence remains a challenge to overcome in order to improve the accuracy and robustness of the automated analysis pipeline.

### Proximity analysis

To quantify the proximity relationships between cell phenotypes we applied HID analysis.^22^ For a pair of cell phenotypes *i*,*j* the HID is calculated to quantify how often these phenotypes occur close to each other in a sample. Let *k*,*l* be cells of of phenotype *i*,*j*, respectively. Then HID is computed as follows:

$$H\left( {i,j} \right) = \left| {\left\{ {\left\{ {{\boldsymbol{x}}_i^k \in C^i,{\boldsymbol{x}}_j^l \in C^j} \right\}\forall \,k,l\,s.t.\left\| {{\boldsymbol{x}}_i^k - {\boldsymbol{x}}_j^l} \right\|_2 < d} \right\}} \right|$$

where ***x*** represents the position of the centroid of a cell and *d* is the parameter that defines closeness. To construct HID we iteratively examine the neighbourhood within a distance *d* around each cell of phenotype *i* and count the number of occurrences of cells of phenotype *j* within that same neighbourhood. The distance parameter *d* is problem specific, as the size of the neighbourhood of interest depends on the type of cells, their mobility and mode of interaction (e.g. directly by contact or indirectly through secretion of cytokines).

The HID measure was normalised using the total number (*N*) of all cells, regardless of phenotype, in samples, as follows:

$$h\left( {i,j} \right) = \frac{{H(i,j)}}{N}$$

The complete image analysis pipeline is presented in Supplementary Fig. 7.

### Statistical analysis

Kaplan–Meier and Proportional Hazards Cox Regression survival analyses for right censored data were performed using the Lifelines 0.18.1 library in Python. Statistical significance of differences between Kaplan–Meier curves was assessed using the Mantel-Haenszel log rank test. The variance of the Kaplan–Meier estimator plotted as error bars in the figures was derived using Greenwood’s formula.^30^ For comparisons of cell distributions between HPV positive and negative subgroups the Mann–Whitney one-sided *U*-test for unpaired data was used. This non-parametric test was selected as the observations did not satisfy the Kolmogorov-Smirnov (K-S) test of normality (*p* < 0.005). Significance is considered at a level *α* = 0.05.

## Results

### Smoking and HPV status predict overall survival

The 72-patient cohort analysed in the current study had a minimum follow up of 7.1 years for the patients who were alive at the time at the time of data collection, and 43 observed events (40% censored data). The median OS of the 72 patients, observed and censored, was 86.8 months. Clinical data for HPV status, stage, alcohol consumption and smoking for the 72 patients are summarised in Supplementary Table 5. Supplementary Table 6 lists the findings from a univariate Cox regression analysis. As expected, negative HPV status was highly prognostic for poor OS (hazard ratio [HR] 3.30; 95% *CI*1.77−6.15; *p* = 0.0002. Smoking also correlated with a worse outcome (*HR* = 1.91, 95% *CI *1.05−3.48, *p* = 0.034).

### Distribution and prognostic value of cell population densities

Table 1 summarises the percent median cell expression of various cell phenotypes in the patient cohort of OPSCC. CD8^+^ T cells and CD68^+^ macrophages were found in significantly greater numbers in HPV^+^ OPSCC tumours. The PD-1^+^ phenotype outnumbered CD8^+^ T cells, which could be explained by PD-1 expression in different T cell subsets, such as CD4^+^ cells. Additionally, the PD-L1^+^ category outnumbered CD68^+^ macrophages, as PD-L1 expression is expected in immune related, as well as tumour cells. These marked populations did not differ significantly when stratified by HPV status. An up to date survival analysis using the median percent marker expression to define high and low expression levels is shown in Table 2. In this study only an increased detection of CD68^+^ cells (macrophages) was significantly associated with improved outcome in the HPV negative patients. It is not possible to compare these results with those published previously as the current analysis did not distinguish the stromal versus tumour locations and a different methodological approach was applied to select ROIs, detect the cells, identify positives and report densities by normalising with total number of cells.^12,15^

**Table 1:** Median population density expressed as a percentage of positive cells^a^

Cell type	All	HPV positive	HPV negative	
T cells	Median percentage of positive cells			*P* value
CD8^+^	6.60%	8.90%	4.90%	0.034
CD8^+^PD-1^+^	1.50%	1.70%	1.10%	0.111
*Macrophages*				
CD68^+^	3.10%	6.00%	2.10%	0.035
CD68^+^PD-L1^+^	1.10%	2.20%	0.70%	0.058
*PD-L1 and PD-1*				
PD-L1^+^	9.00%	9.00%	7.40%	0.356
PD-1^+^	12.70%	13.50%	10.90%	0.284

^a^Percentage cell expression was first assessed for individual ROIs, and the median expression from all ROI was selected to represent the patient

**Table 2:** Univariate Cox Regression analysis of overall survival for patients stratified by median cell expression^a^

	HPV positive		HPV negative		All	
Cell population	HR (95% CI)	P value	HR (95% CI)	*P* value	HR (95% CI)	*P* value
*T cells*						
CD8^+^	0.56 (0.21, 1.50)	0.25	1.03 (0.47, 2.26)	0.94	0.84 (0.46, 1.54)	0.57
CD8^+^PD-1^+^	0.76 (0.29, 1.99)	0.57	1.88 (0.85, 4.16)	0.12	1.16 (0.63, 2.14)	0.63
*Macrophages*						
CD68^+^	1.34 (0.51, 3.53)	0.55	0.34 (0.14, 0.79)	0.01	0.58 (0.32, 1.07)	0.08
CD68^+^PD-L1^+^	1.33 (0.51, 3.45)	0.56	1.50 (0.67, 3.34)	0.32	1.34 (0.73, 2.47)	0.34
*PD-L1 and PD-1*						
PD-L1^+^	1.36 (0.52, 3.52)	0.53	1.50 (0.67, 3.34)	0.32	1.42 (0.77, 2.60)	0.26
PD-1^+^	0.60 (0.22, 1.60)	0.31	1.84 (0.83, 4.07)	0.14	1.06 (0.57, 1.94)	0.86

^a^Variables stratified by the median to distinguish patients with high and low expression. Percentage cell expression was first assessed for individual ROIs, and the median expression from all ROI was selected to represent the patient

### Proximity analyses of T cells with PD-L1^+^ cells

**Fig. 1:**
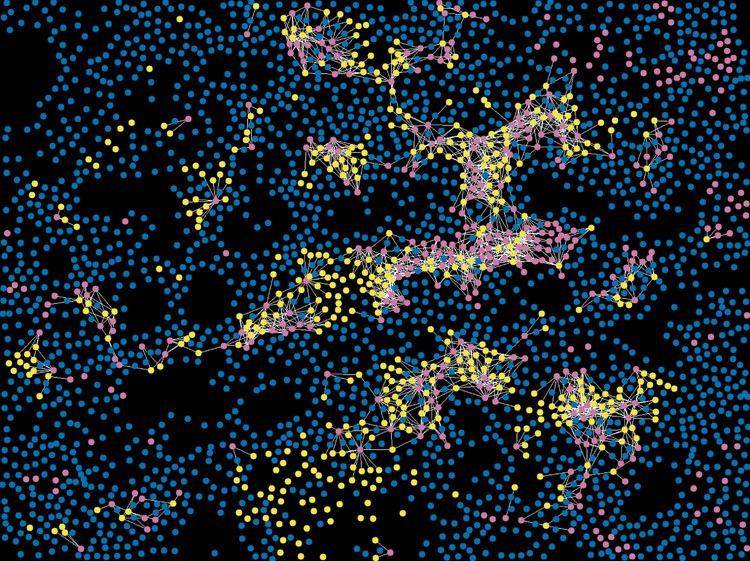
Illustrative HID interaction features for a region of interest. An interaction is hypothesised to occur whenever a CD8^+^ cell (yellow) occurs within 30 μm of a PD-L1^+^ cell (pink). A connection is drawn (white) to represent each hypothesised interaction. Cells not expressing CD8 or PD-L1 are presented in blue

**Fig. 2:**
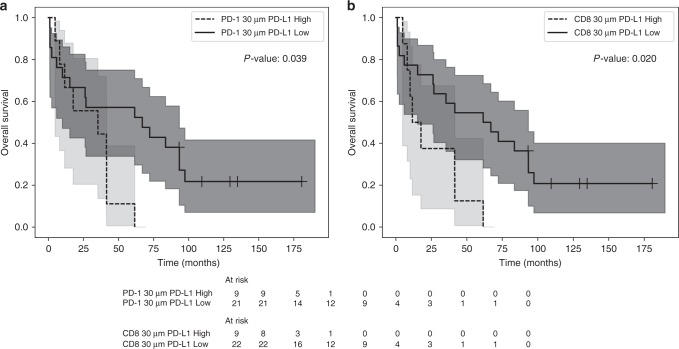
Kaplan–Meier analysis of the effect of HID interactions on prognosis in the HPV negative subgroup. Significance is considered using the log rank test. High and low co-localisations are considered by splitting the patients at the mean value. **a** Interactions between PD-L1^+^ and PD-1^+^ cells. **b** Interactions between PD-L1^+^ and CD8^+^ cells

Figure 1 illustrates an example of the methodology used to generate the HID measure reflecting potential cell interactions. An interaction is hypothesised to occur whenever a CD8^+^ cell (yellow) occurs within 30 μm of a PD-L1^+^ cell (pink). A connection is drawn (white) to represent each hypothesised interaction. Cells not expressing CD8 or PD-L1 are presented in blue. A pre-specified HID analysis was carried out for two pairs of interacting phenotypes co-localised within 30 μm of each other (CD8^+^ and PD-L1^+^ cells; PD-1^+^ and PD-L1^+^ cells). This distance was used by Feng et al.^21^ and represents a neighbourhood size of 2–3 cells. The mean ± SEM HID values are shown in Table 3. There was a larger number of CD8/PD-L1 and PD-1/PD-L1 proximal events in the HPV positive tumours, but the difference was not statistically significant. A univariate Cox regression analysis stratified patients by high versus low levels of co-localisation (percent mean). More frequent interactions between CD8^+^ and PD-L1^+^ or PD-1^+^ and PD-L1^+^ cells were prognostic for poor overall survival in HPV^-^ but not HPV^+^ patients or the whole cohort (Table 4, Fig. 2). When stratifying the HPV^−^ patients by the mean value of PD-1^+^ and PD-L1^+^ HID interactions, 30% of the patients were assigned to the poor prognostic group. When grouping by CD8^+^ and PD-L1^+^ interactions, 23% of patients were assigned to the poor prognostic group (Fig. 2a, b).

**Table 3:** Distribution of HID features in all, HPV positive and HPV negative patients^a^

Cell interactions	All	HPV positive	HPV negative	*P* value
CD8^+^ within 30 μm of PD-L1^+^	27.65 (±6.86)	34.73 (±10.68)	17.73 (±6.69)	0.276
PD-1^+^ within 30 μm of PD-L1^+^	15.76 (±7.32)	23.48 (±12.41)	4.95 (±1.72)	0.535

^a^The mean ± (standard error) for all, HPV positive and HPV negative patients. Units given as: density of interactions · 10^3^

**Table 4:** Univariate Cox Regression analysis of overall survival for patients stratified by mean HID proximity frequencies

	HPV positive		HPV negative		All	
Cell interactions	HR (95% CI)	*P* value	HR (95% CI)	*P* value	HR (95% CI)	*P* value
CD8^+^ within 30 μm of PD-L1^+^	0.82 (0.26, 2.50)	0.73	2.95 (1.15, 7.56)	0.02	1.15 (0.58, 2.30)	0.68
PD-1^+^ within 30 μm of PD-L1^+^	0.59 (0.17, 2.06)	0.41	2.64 (1.04, 6.71)	0.04	1.15 (0.58, 2.29)	0.69

## Discussion

This study introduces an automated pipeline for analysis of different biomarkers in the tumour micro-environment. In comparison with other automated image analysis studies, our pipeline used an automated quality check of scanned images and ROI selection prior to quantification of spatial interaction features. Checking image quality is a time consuming but essential part of any histopathological analysis. Blurred areas and artefacts (e.g. bubbles, tissue folds, presence of fatty tissue) lead to processing errors and consequently the samples are sent back for re-staining and scanning, increasing the time required for analysis. This automated selection of good quality ROIs decreases the need for input from a pathologist. A key component of this study is the use of HID methodology which can be used to assess the spatial relations (proximity) between particular cell phenotypes.^22^ It has previously been used by us to analyse T cell regulatory patterns in follicular lymphoma.^31,32^

Our study provides novel evidence that the frequent proximity of PD-1^+^ and PD-L1^+^ cells is an adverse prognostic factor in HPV^-^ OPSCC. It is tempting to speculate this derives from the functional consequence of these interactions in the PD-1/PD-L1 pathway of immune escape. If the latter is correct, then quantifying the frequency of proximal cell-cell interactions using HID should be further explored as a secondary companion diagnostic potentially useful in directing checkpoint inhibitor treatment. Monitoring levels of PD-L1 expression alone, while biologically plausible, has shown inconsistent results, particularly in cases where expression levels are close to the cut-off threshold.^13^ Interestingly, in our analysis we observed no correlation between PD-L1 expression and overall survival, regardless of HPV status for OPSCC. This result agrees with the observations from other studies.^16,21^ However, previous analyses of the same cohort^15^ demonstrated that PD-L1 expression was prognostic in HPV negative OPSCC but only if assessed in the stromal regions with a cut-off of 5%. The optimal manner of scoring PD-L1 is still being investigated, as the cut-off thresholds differ in lung, urothelial and head and neck cancer. Indeed, opinions differ on whether positivity should be assessed only for tumour cells or additionally for immune infiltrating cells.^33^ An automated process to quantifying cell patterns promotes consistency and reproducibility and could facilitate its use to support the role of PD-L1 in personalised treatment strategies.

Interestingly, the correlation between overall survival and HID spatial interactions in the HPV positive subgroup was not significant. If this is a true effect, it would indicate reduced importance of T and PD-L1^+^ cell interactions for the HPV^+^ subgroup. This finding is not surprising as HPV related OPSCC is considered in many aspects different from HPV^-^ OPSCC and is known to have a better prognosis,^34^ more active anti-tumour immune response^12^ and favourable response to treatment.^34^ However, the nature of PD-L1^+^ spatial interactions in HPV^+^ OPSCC merits further investigation in larger cohorts, before their significance could be ruled out.

Due to the size of the cohort the power of the study is limited which increases the risk of false negative results. To avoid multiple testing, we only explored two pre-determined hypotheses using HID in relation to overall survival. Another possible limitation is the image analysis pipeline, which involved a single pathologist identifying positive cells by selecting a cut-off for each marker based on selected images with clear positive staining. However, variation was observed between the intensities of positive cells in different sections, which a simple on-off scoring approach cannot capture. Accuracy in scoring could be more reliable if it was carried out using ground truth either from multiple pathologists, a complementary modality, such as transcriptomics or flow cytometry, or an index tissue microarray section with cores constructed from cell lines of positive and negative cells used as reference.

In summary, our study combined multiplex immunofluorescence and multispectral microscopy with an automated analysis pipeline for quality checking, spectral unmixing, cell segmentation, scoring and assessment of the spatial pattern of cell-cell interactions. In a cohort of OPSCC patients we showed that frequent proximity of CD8^+^ or PD-1^+^ and PD-L1^+^ cells was prognostic for OS in patients with HPV^−^ tumours. Our method is ready to be tested independently in additional, multicentre cohorts to validate its potential as a companion diagnostic for therapies targeting the PD-1/ PD-L1 pathway of immune escape.

## Supplementary information

Supplementary Material

## Data Availability

Data and programming scripts are available from the corresponding author upon reasonable request.

## References

[1] Galon J, Mlecnik B, Bindea G, Angell HK, Berger A, Lagorce C (2014). Towards the introduction of the ‘Immunoscore’ in the classification of malignant tumours. J. Pathol..

[2] Schreiber RD, Old LJ, Smyth MJ (2011). Cancer immunoediting: integrating the role of immunity in cancer suppression and promotion. Science.

[3] Wang D, Du Bois RN (2015). Immunosuppression associated with chronic inflammation in the tumor microenvironment. Carcinogenesis.

[4] Pardoll DM (2012). The blockade of immune checkpoints in cancer immunotherapy. Nat. Rev. Cancer.

[5] Ribas A, Wolchok JD (2018). Cancer immunotherapy using checkpoint blockade. Science.

[6] Topalian SL (2017). Targeting immune checkpoints in cancer therapy. Jama.

[7] Chae YK, Arya A, Iams W, Cruz MR, Chandra S, Choi J (2018). Current landscape and future of dual anti-CTLA4 and PD-1/PD-L1 blockade immunotherapy in cancer; lessons learned from clinical trials with melanoma and non-small cell lung cancer (NSCLC). J. Immunother. Cancer.

[8] Ghatalia P, Zibelman M, Geynisman DM, Plimack ER (2017). Checkpoint inhibitors for the treatment of renal cell carcinoma. Curr. Treat. Options Oncol..

[9] Zolkind P, Uppaluri R (2017). Checkpoint immunotherapy in head and neck cancers. Cancer Metastasis Rev..

[10] Ran X, Yang K (2017). Inhibitors of the PD-1/PD-L1 axis for the treatment of head and neck cancer: current status and future perspectives. Drug Des. Devel. Ther..

[11] Solomon B, Young RJ, Rischin D (2018). Head and neck squamous cell carcinoma: genomics and emerging biomarkers for immunomodulatory cancer treatments. Semin. Cancer Biol..

[12] Oguejiofor K, Hall J, Slater C, Betts G, Hall G, Slevin N (2015). Stromal infiltration of CD8 T cells is associated with improved clinical outcome in HPV-positive oropharyngeal squamous carcinoma. Br. J. Cancer.

[13] Shen X, Zhao B (2018). Efficacy of PD-1 or PD-L1 inhibitors and PD-L1 expression status in cancer: meta-analysis. BMJ.

[14] Müller T, Braun M, Dietrich D, Aktekin S, Höft S, Kristiansen G (2017). PD-L1: a novel prognostic biomarker in head and neck squamous cell carcinoma. Oncotarget.

[15] Oguejiofor K, Galletta-Williams H, Dovedi SJ, Roberts DL, Stern PL, West CML (2017). Distinct patterns of infiltrating CD8+ T cells in HPV+ and CD68 macrophages in HPV - oropharyngeal squamous cell carcinomas are associated with better clinical outcome but PD-L1 expression is not prognostic. Oncotarget.

[16] Schneider S, Kadletz L, Wiebringhaus R, Kenner L, Selzer E, Füreder T (2018). PD-1 and PD-L1 expression in HNSCC primary cancer and related lymph node metastasis - impact on clinical outcome. Histopathology.

[17] Ou D, Adam J, Garberis I, Blanchard P, Nguyen F, Levy A (2017). Clinical relevance of tumor infiltrating lymphocytes, PD-L1 expression and correlation with HPV/p16 in head and neck cancer treated with bio- or chemo-radiotherapy. Oncoimmunology.

[18] Feichtenbeiner A, Haas M, Büttner M, Grabenbauer GG, Fietkau R, Distel LV (2014). Critical role of spatial interaction between CD8+ and FOXP3+ cells in human gastric cancer: the distance matters. Cancer Immunol. Immunother..

[19] Spagnolo DM, Gyanchandani R, Al-Kofahi Y, Stern AM, Lezon TR, Gough A (2016). Pointwise mutual information quantifies intratumor heterogeneity in tissue sections labeled with multiple fluorescent biomarkers. J. Pathol. Inform..

[20] Scognamiglio T, Chen Y-T (2018). Beyond the percentages of PD-L1-positive tumor cells: induced versus constitutive PD-L1 expression in primary and metastatic head and neck squamous cell carcinoma. Head Neck Pathol..

[21] Feng Z, Bethmann D, Kappler M, Ballesteros-Merino C, Eckert A, Bell RB (2017). Multiparametric immune profiling in HPV– oral squamous cell cancer. JCI Insight.

[22] Rose CJ, Naidoo K, Clay V, Linton K, Radford JA, Byers RJ (2013). A statistical framework for analyzing hypothesized interactions between cells imaged using multispectral microscopy and multiple immunohistochemical markers. J. Pathol. Inform..

[23] Tóth ZE, Mezey É (2007). Simultaneous visualization of multiple antigens with tyramide signal amplification using antibodies from the same species. J. Histochem. Cytochem..

[24] Dickinson ME, Bearman G, Tille S, Lansford R, Fraser SE (2001). Multi-spectral imaging and linear unmixing add a whole new dimension to laser scanning fluorescence microscopy. Biotechniques.

[25] Kohlberger T., Liu Y., Moran M., Po-Hsuan, Chen, Brown T et al. Whole-slide image focus quality: automatic assessment and impact on ai cancer detection. Preprint at: http://arxiv.org/abs/1901.04619 (2019). 10.4103/jpi.jpi_11_19 PMC6939343 31921487

[26] Hossain M. S., Kimura F., Yagi Y., Yamaguchi M., Nakamura T. Practical image quality evaluation for whole slide imaging scanner. in SPIE Proc. *Structured Light Conference: Biomedical Imaging Sensing Conference*, SPIE Publications, 107111S (2018).

[27] Ronneberger O., Fischer P., Brox T. U-net: Convolutional networks for biomedical image segmentation. in Proc. *International Conference Med Image Computing Comput Intervention*. Springer, Cham, 234–241 (2015).

[28] Bankhead P, Loughrey MB, Fernández JA, Dombrowski Y, Mcart DG, Dunne PD (2017). QuPath: open source software for digital pathology image analysis. Sci Rep.

[29] Schmidt U., Weigert M., Broaddus C., Myers G. Cell detection with star-convex polygons. in Proc. International Conference Med Image Computing Comput Intervention. 265–273 (2018).

[30] Greenwood, M. Reports on Public Health and Medical Subjects No. 33. A Report on the Natural Duration of Cancer. iv–26. (H.M.S.O.: London, 1926).

[31] Nelson LS, Mansfield JR, Lloyd R, Oguejiofor K, Salih Z, Menasce LP (2015). Automated prognostic pattern detection shows favourable diffuse pattern of FOXP3+ Tregs in follicular lymphoma. Br J Cancer.

[32] Tsakiroglou A. M., Fergie M., West C., Linton K., Astley S., Byers R. et al. Quantifying cell-type interactions and their spatial patterns as prognostic biomarkers in follicular lymphoma. in SPIE Proc. *Medical Imaging Conference: Digital Pathology*, SPIE Publications, 105810G (2018).

[33] Herbst RS, Soria JC, Kowanetz M, Fine GD, Hamid O, Gordon MS (2014). Predictive correlates of response to the anti-PD-L1 antibody MPDL3280A in cancer patients. Nature.

[34] Kobayashi K, Hisamatsu K, Suzui N, Hara A, Tomita H, Miyazaki T (2018). A review of HPV-related head and neck cancer. J. Clin. Med..

