# Supplementary material for: Spatial proximity between T and PD-L1 expressing cells as a prognostic biomarker for oropharyngeal squamous cell carcinoma
Source: Br J Cancer. 2019 Dec 6;122(4):539–44. doi: 10.1038/s41416-019-0634-z (PMC7028988; doi:10.1038/s41416-019-0634-z)
Supplement: Supplementary file 1 — Supplementary Material [file 41416_2019_634_MOESM1_ESM.docx]

# Multiplex Immunofluorescent Staining Protocol

**Table 1.** Antibodies, titrations and fluorophores in the multiplex immune-fluorescent experiment. The order presented reflects the order in which the antibodies were placed on the tissue.

| Order | Antibody | Dilution | Provider | Opal detection |
| --- | --- | --- | --- | --- |
| 1 | Rabbit monoclonal against PD-L1 | 1:200 | Cell Signalling, US | Cyanine 5.5 |
| 2 | Mouse monoclonal against CD8 (clone C8/144B) | 1:60 | Dako, Denmark | Cyanine 3 |
| 3 | Mouse monoclonal against PD1 | 1:50 | Abcam, UK | Fluorescein |
| 4 | Mouse monoclonal against CD68 | 1:200 | Abcam, UK | Cyanine 3.5 |


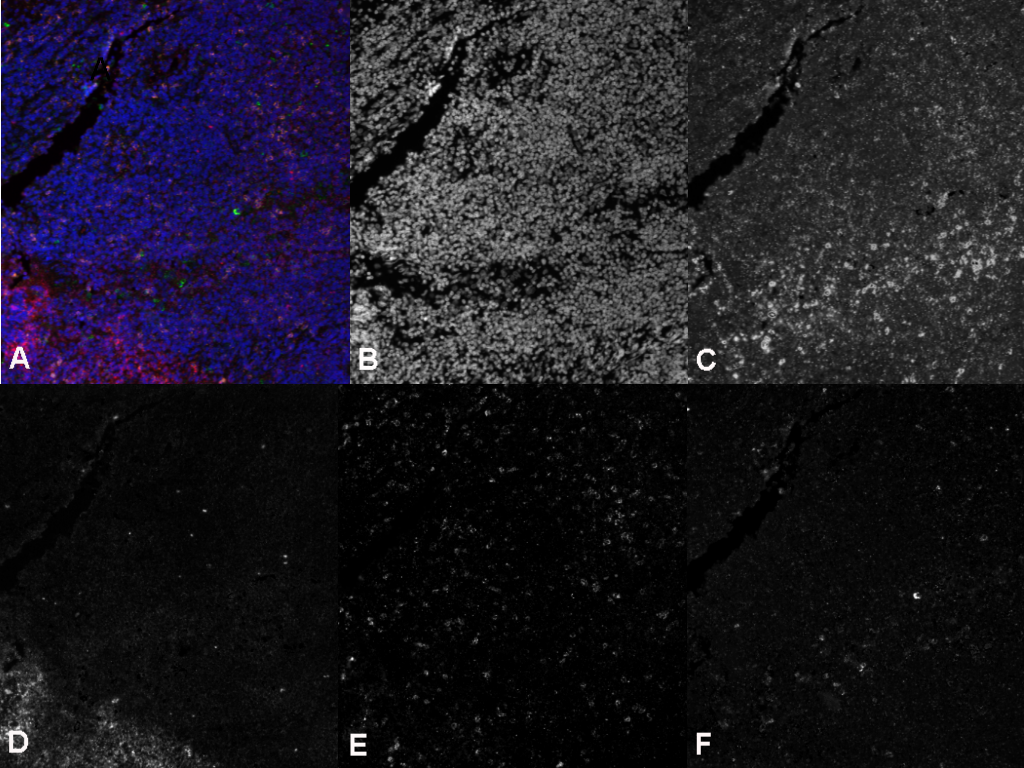


**Figure 1.** Example of an image in the data set, representing a single region of interest (1040 ×1392 pixels). A: Composite view where DAPI, CD8, CD68, PD-L1 and PD-1 were mapped to blue, green, yellow, red and magenta, respectively; B: DAPI nuclear counter-stain; C: PD-1; D: PD-L1; E: CD68 and F: CD8. The image also includes a channel with tissue auto-fluorescence (AF) signal - not shown here. AF has already been subtracted from all other channels during spectral un-mixing.

# Deep Learning Architecture for Automated Image Quality Check

32^2^

16^2^

32^2^

16^2^

16^2^

Convolution 3x3, eLU

Concatenate

Max pool 2x2

Up-convolution 3x3, eLU

Convolution 1x1, softmax

6 16 16

16 32 32

32 64 64

64 128 128

128 256 256

256 128 128

128 64 64

64 32 32

32 16 16 3

128^2^

128^2^

128^2^

64^2^

64^2^

64^2^

32^2^

32^2^

32^2^

32^2^

64^2^

64^2^

64^2^

128^2^

128^2^

128^2^

128^2^

8^2^

8^2^

8^2^

16^2^

16^2^

16^2^

**Figure 2.** The architecture of the U-net segmentation model.

Ground truth annotations were drawn using the open-source software QuPath and exported automatically using custom Groovy scripts, as this platform allows the user to easily implement their own algorithms to supplement the functionality of the main interface. Are available upon request. This implementation allowed us to keep the full resolution of the image and assign an integer label to each pixel based on the drawn annotation (0: background or fatty tissue, 1: useful tissue, 2: artefacts). The annotations were created by observing all the stains separately and also the composite image. The ROI images were tiled into patches of size $128\times128\times6$, run through the network and a map of the predicted pixel labels was generated for each patch (as in Supplementary Figure 3). The predicted pixel labels from all patches were merged to create the map of the entire ROI. Tiling greatly improved the memory requirements for the task.

Keras with a Tensforflow backend was used for the implementation and training was performed on the Collaboratory servers for 200 epochs and a batch size of 20. The Adaptive Moment Estimation (Adam) optimiser was used with the categorical cross-entropy loss and a learning rate parameter of 0.0005. All image channels were included during training. A separate validation set of 960 images (size 128×128×6) was used to tune empirically the hyper parameters of the network by observing the validation loss. The pixel-wise predicted accuracy on the validation set was 92.9% and 88.3% on the test set.

**Table 2**. Normalised confusion matrix for the network predictions on the test set.

|  |  | **U-net CNN Confusion Matrix** | | |
| --- | --- | --- | --- | --- |
| True label | Tissue | 0.92 | 0.06 | 0.02 |
|  | Artefact | 0.22 | 0.75 | 0.03 |
|  | Background | 0.01 | 0.10 | 0.89 |
|  |  | Tissue | Artefact | Background |
|  |  | Predicted label | | |


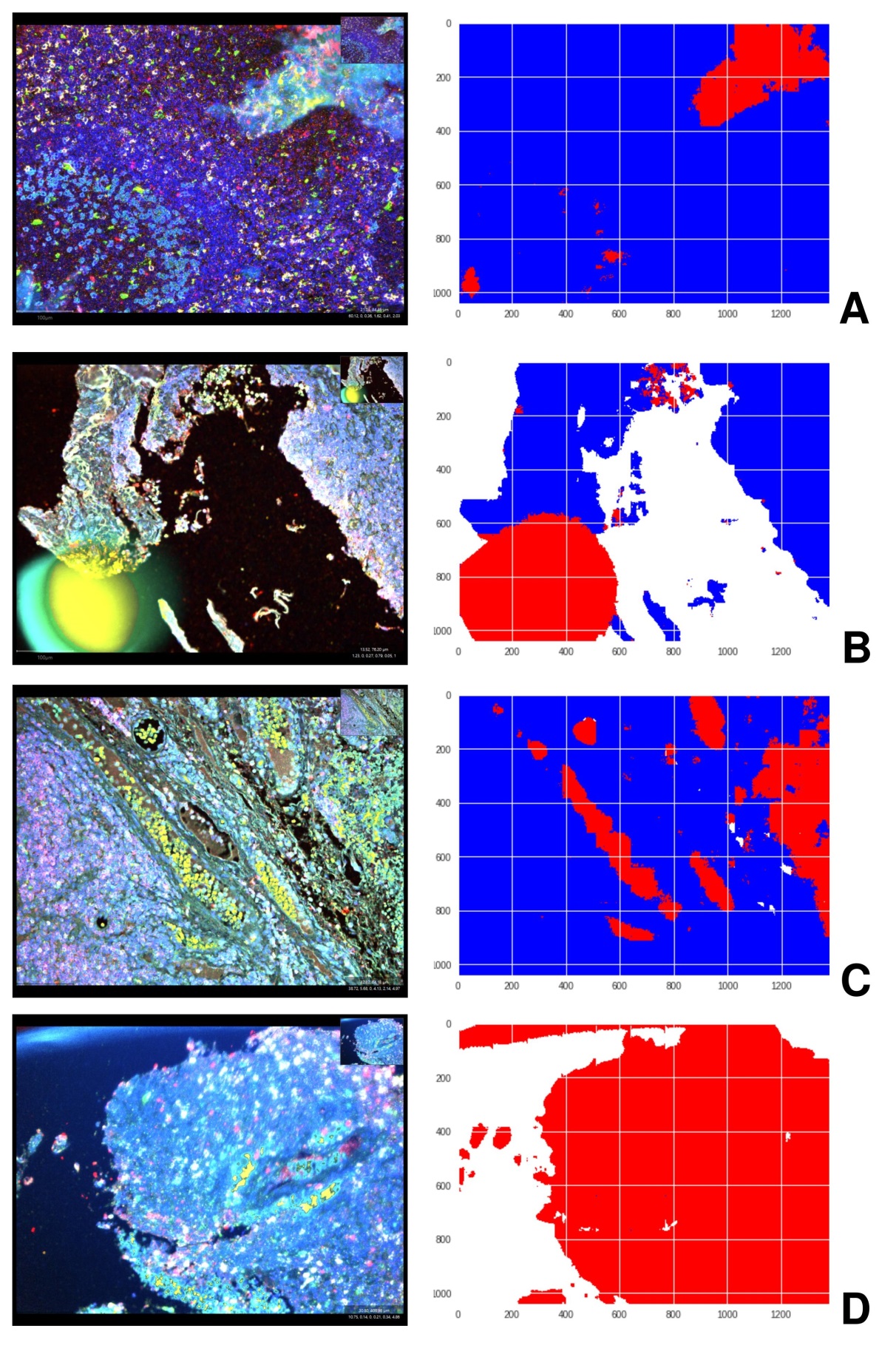


**Figure 3** Problematic areas and predicted segmentation labels from the test set. A. Area with tissue fold (upper right corner). B. Bubble due to poor cover-slipping. C. Blood vessels filled with red blood cells. D. Whole region scanned out of focus. The colour-map of predicted segmentation masks (images on the right) as follows; red: problematic area, blue: normal tissue, white: background.

We compared the performance of our own automated artefact finder using U-net to the image segmentation algorithm supplemented in inForm 2.4 (Akoya Biosciences) software. For that purpose the same set of manually drawn full image annotations previously used to train U-net, was also used to train a tissue segmentation module in inForm. The training was allowed to continue, until the training accuracy stabilised at 91%, while the segmentation resolution was set to coarse. All fluorophore channels and auto-fluorescence were used during training. To assess performance, the images of the test set were then processed using the trained inForm algorithm, resulting in pixel-wise accuracy of 81.2%, which was markedly worse compared to the 88.3% accuracy obtained by the U-net classifier for the same test set.

# Cell Segmentation and Scoring

To assess the nuclear segmentation accuracy, a manually annotated set of 5 ROI, containing 956 nuclei in total, was constructed from the head and neck data set. We compared the performance of unsupervised nuclear segmentation in inForm 2.4 and unsupervised nuclear segmentation using a watershed algorithm in QuPath 0.1.3 open-source software. QuPath was used to generate and export the manual segmentation annotations as labelled integer masks by using custom Groovy scripting.

To compare these two algorithms we followed Schmidt et al.^29^ and calculated the average precision$AP= \frac{TP}{TP+FP+FN}$, where true positive (TP) predictions are defined as predicted nuclei which sufficiently overlap annotated ground truth (GT) nuclei. Overlap was measured as the intersection over union (IoU) between predicted and ground truth cells, but as “sufficient” overlap can be tricky to define, we repeated the performance assessment for various thresholds of the IoU metric. False positives (FP) were defined as the predicted nuclei with no corresponding ground truth nuclei, while false negative (FN) were the ground truth nuclei with no corresponding predicted nuclei.

Many different settings for algorithm implementation in inForm were qualitatively tested through trial and error, and the optimal settings were selected, as presented in Supplementary Table 3. The average precision results, calculated on a cell-wise basis, are presented in Supplementary Table 4. Supplementary Figure 4 demonstrates the segmentation results for each ROI. According to these experiments, the nuclear segmentation performance of QuPath and inForm in the manually annotated test set is approximately equal, and the predicted nuclear masks are similar.

**Table 3.** Nuclear segmentation settings for inForm 2.4 and QuPath 0.1.3.

| **Settings for inForm 2.4** | |
| --- | --- |
| Algorithm | Adaptive cell segmentation |
| Component | DAPI |
| Relative intensity threshold | 0.1 |
| Nuclear staining quality | Mixed |
| Nuclear splitting settings threshold | 0.442 |
| Minimum nuclear size (pixels) | 60 |
| Fill nuclear holes smaller than (pixels) | 50 |
| Refining cell after segmentation | FALSE |
| **Settings for QuPath 0.1.3** | |
| Algorithm | Watershed cell detection |
| Component | DAPI |
| Requested pixel size | 0.5 |
| Background radius | 8 |
| Median filter radius | 0.8 |
| Sigma | 1.2 |
| Minimum area (μm^2^) | 5 |
| Maximum area (μm^2^) | 200 |
| Intensity threshold | 2 |
| Split by shape | TRUE |
| Smooth boundaries | TRUE |

**Table 4.** Segmentation performance in the manually annotated test set. AP is the average precision for different values of the intersection over union threshold (IoU). As the IoU threshold increases the definition of a true positive cell becomes stricter (i.e. the shape of the predicted cell must match more closely the ground truth).

| Intersection over union (IoU) threshold | 10% | 20% | 30% | 40% |
| --- | --- | --- | --- | --- |
| AP inForm 2.4 | 0.845 | 0.755 | 0.65 | 0.495 |
| AP QuPath 0.1.3 | 0.865 | 0.762 | 0.634 | 0.51 |


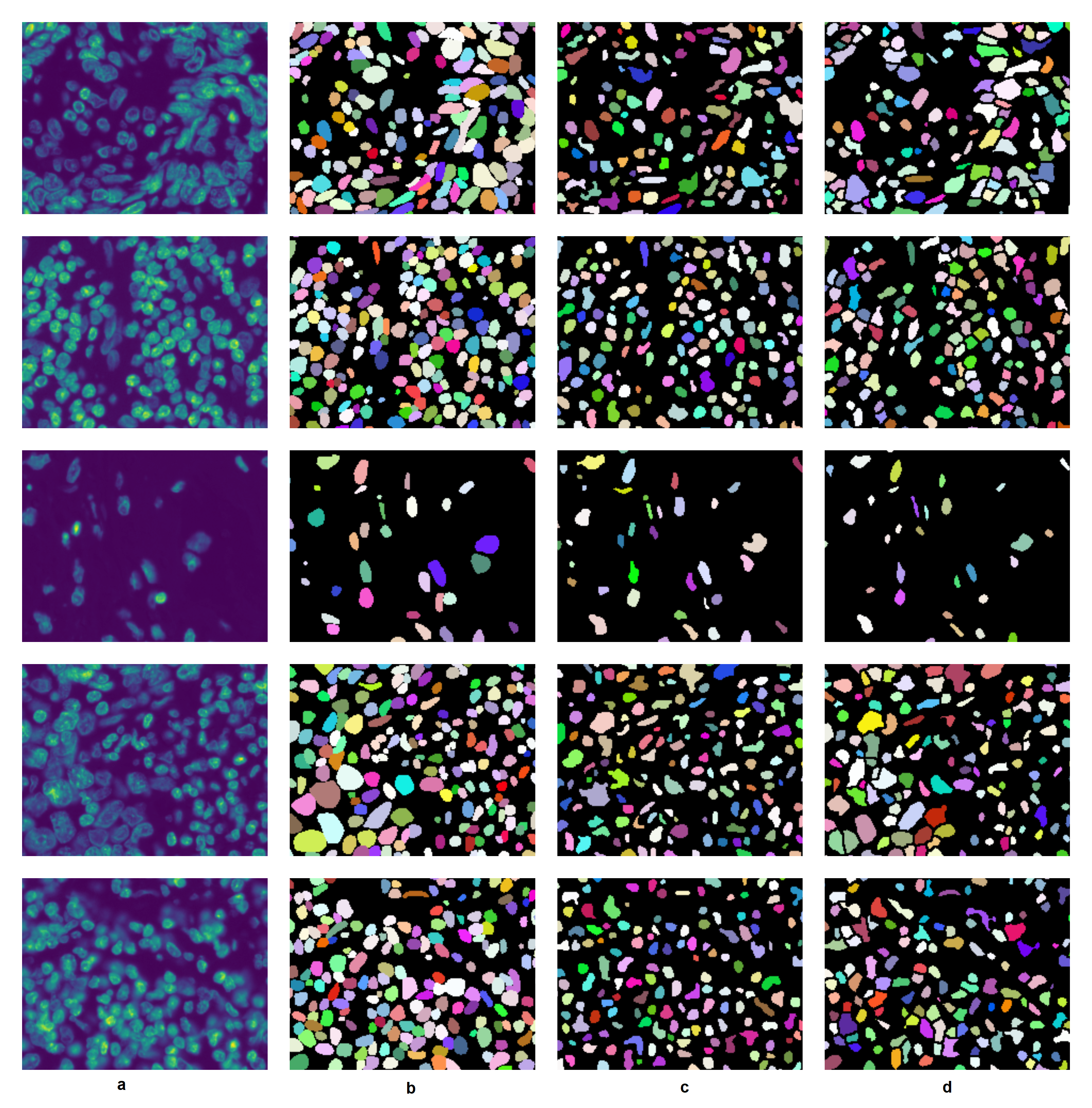


**Figure 4.** Nuclear segmentation comparison between inForm 2.4 and QuPath 0.1.3. a) DAPI component b) manual annotations c) QuPath segmentation results d) inForm segmentation results.


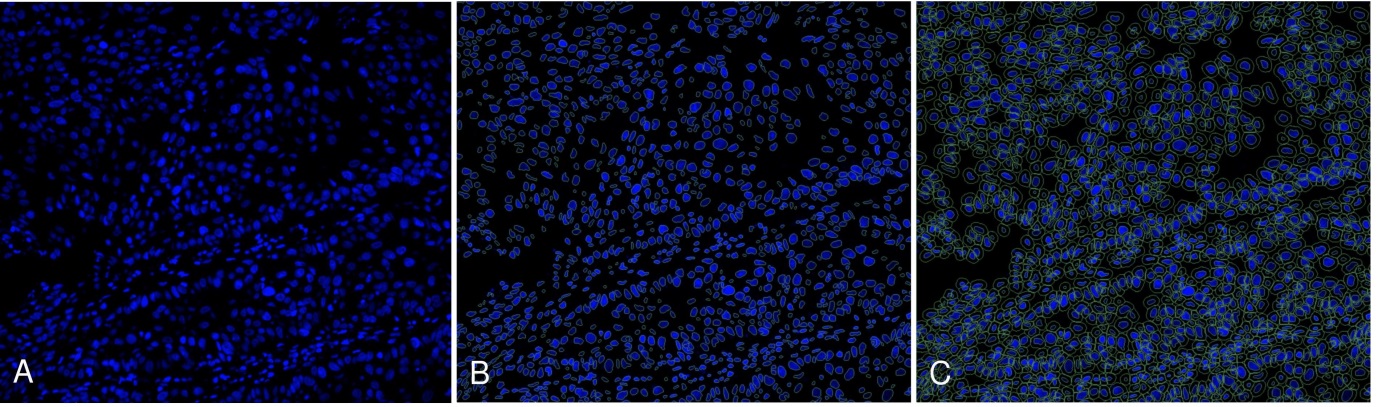


**Figure 5**. Cell segmentation was carried out in QuPath. A. DAPI channel view; B. Nucleus segmentation; C. Cytoplasm simulation by nucleus expansion. A Groovy batch script was written using QuPath’s interface to segment the cells in all images and export the cell data


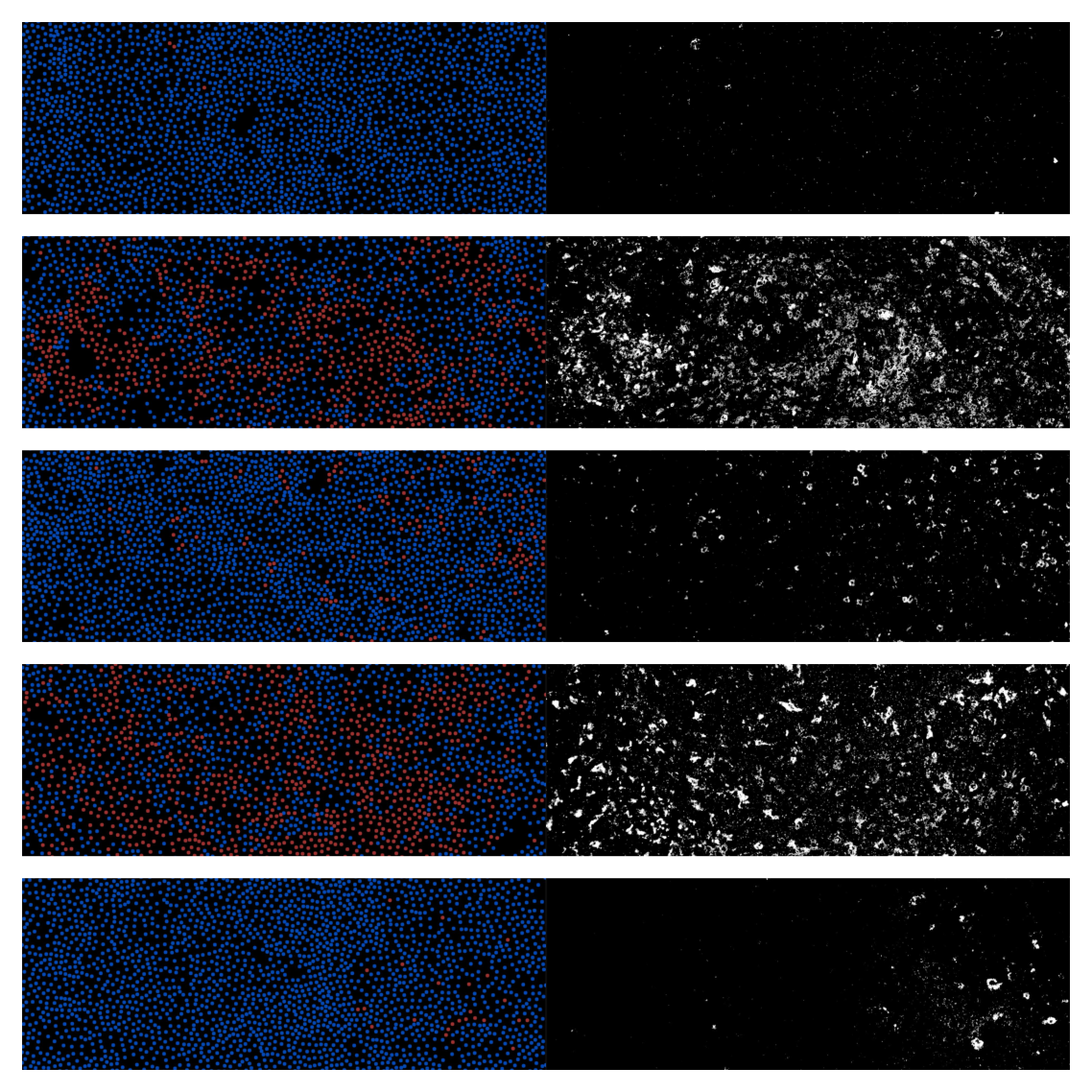


**Figure 6.** Results of scoring for five regions of interest (ROI) from different slides and patients for the CD8 marker. These images were part of the set used when selecting the thresholds. The colour map of the grey scale images is scaled to range between the minimum and maximum intensity value for CD8 in the entire data set.


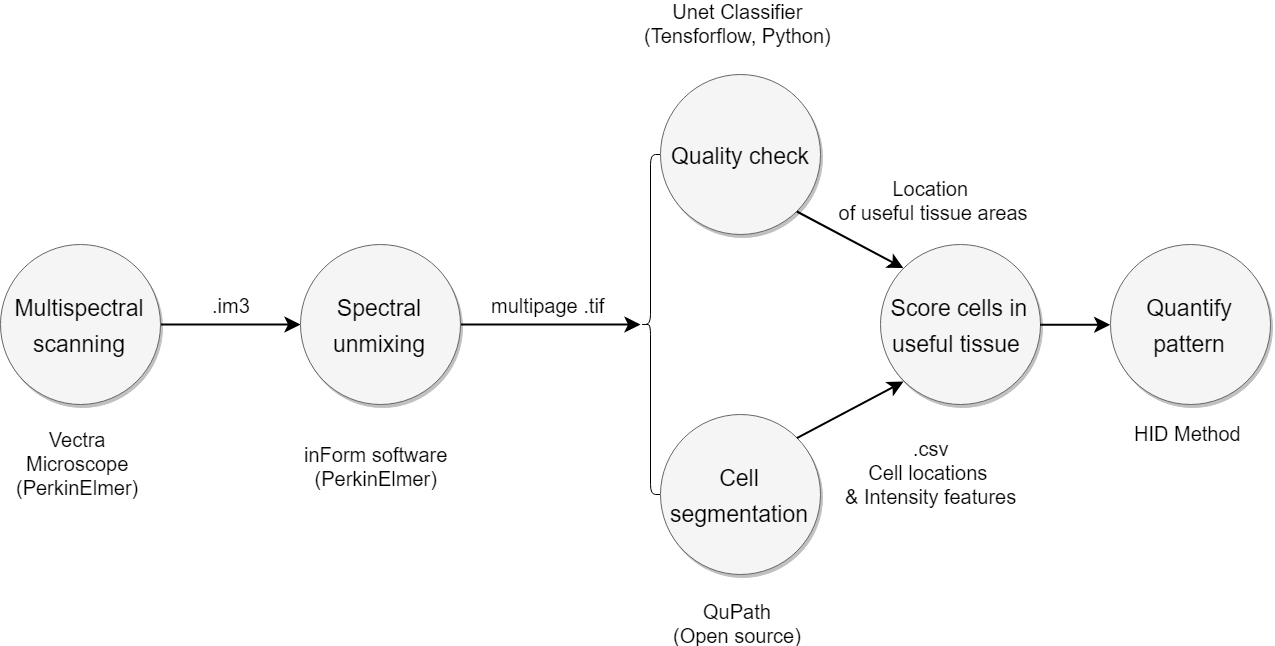


**Figure 7.** Diagram of image analysis pipeline.

# Cohort Characteristics

**Table 5.** Cohort characteristics.

| Characteristic | | All | HPV Positive | HPV Negative |
| --- | --- | --- | --- | --- |
| Patients [Events Observed OS] |  | 72 [43] | 41 [18] | 31 [25] |
| Gender | Female | 12 | 7 | 5 |
|  | Male | 60 | 34 | 26 |
| Age (years) | Median | 58 | 56 | 59.5 |
| AJCC Stage | I | 0 | 0 | 0 |
|  | II | 2 | 1 | 1 |
|  | III | 10 | 4 | 6 |
|  | IV | 23 | 14 | 9 |
|  | No data | 37 | 22 | 15 |
| Grade | Well differentiated | 10 | 9 | 1 |
|  | Moderately differentiated | 36 | 13 | 23 |
|  | Poorly differentiated | 19 | 14 | 5 |
|  | No data | 7 | 5 | 2 |
| Alcohol | Never | 8 | 5 | 3 |
|  | Moderate | 35 | 19 | 16 |
|  | Excessive | 27 | 16 | 11 |
|  | No data | 2 | 1 | 1 |
| Smoking | Never | 14 | 12 | 2 |
|  | Ex-smoker | 29 | 16 | 13 |
|  | Current smoker | 27 | 12 | 15 |
|  | No data | 2 | 1 | 1 |

# Prognostic Value of Clinical Variables

**Table 6.** Cox regression survival analysis (univariate) for clinical variables^*^.

|  | HR (CI 95%) | P value |
| --- | --- | --- |
| HPV status | 3.295 (1.767, 6.145) | *0.0002* |
| AJCC stage | 0.790 (0.396, 1.579) | 0.5052 |
| Alcohol | 0.982 (0.760, 1.269) | 0.8896 |
| Smoking | 1.911 (1.049, 3.481) | *0.0342* |
| Grade | 0.979 (0.636, 1.507) | 0.9226 |
| ^*^Alcohol was assessed as 0: never, 1: moderate and 2: excessive, while smoking was assessed as 0: never or ex-smoker and 1: currently smoking. Grade was given as 0: well differentiated, 1: moderately and 2: poorly and stage as I-IV according to staging criteria set by the American Joint Committee on Cancer (AJCC). | | |
